# Precision Livestock Farming and Biomedical Engineering: Assessing Feed Quality, Animal Health, and Behavior Using Machine Learning for Sensor Data

**DOI:** 10.3390/s26134015

**Published:** 2026-06-24

**Authors:** Nikolay Kiktev, Danylo Hradoboiev, Mykola Pravilov, Ievgen Antypov, Yuliia Meish, Liliia Stroianovska, Pawel Kielbasa, Taras Hutsol

**Affiliations:** 1 Department of Automation and Robotic Systems, National University of Life and Environmental Sciences of Ukraine, 15 Heroiv Oborony Str., 03041 Kyiv, Ukraine; d.gradoboev@nubip.edu.ua (D.H.); kolyapra@ukr.net (M.P.); 2 Department of Power Systems Engineering, National University of Life and Environmental Sciences of Ukraine, 15 Heroiv Oborony Str., 03041 Kyiv, Ukraine; ievgeniy.antypov@nubip.edu.ua; 3 Department of Higher and Applied Mathematics, National University of Life and Environmental Sciences of Ukraine, 15 Heroiv Oborony Str., 03041 Kyiv, Ukraine; juliameish@nubip.edu.ua; 4 Department of Animal Hygiene and Veterinary Support of the Cynological Service of the National Police of Ukraine, Higher Educational Institution “Podillia State University”, 32-300 Kamianets-Podilskyi, Ukraine; liliiastroianovska18@gmail.com; 5 Department of Machine Operation, Ergonomics and Production Processes, Faculty of Production and Power Engineering, University of Agriculture in Krakow, Balicka 116B, 30-149 Krakow, Poland; pawel.kielbasa@urk.edu.pl; 6 Ukrainian University in Europe—Foundation, Balicka 116, 30-149 Krakow, Poland

**Keywords:** accelerometry, spectrometry, tensometry, biosensorics, videomonitoring, telemetry, datastream, CNN, ML, DL, animals, diagnostics, premix

## Abstract

This review analyses and logically structures modern intelligent sensor technologies in the context of animal husbandry, feed production, and veterinary medicine. The main research discussed in the article focuses on machine learning based on modern neural network models, computer vision, and sensor systems that are transforming the methods for assessing the health, behavior, and nutrition of farm animals. The first part examines modern approaches to quality control and optimization of mineral and vitamin premixes, including visual inspection using visual sensors and neural networks. Key roles are played by precise dosing, component stability (minerals, vitamins), and the transition to more bioefficient organic forms of micronutrients to reduce environmental impact. Improvements in feed and premix production are analyzed, including automation, energy management, and the use of machine learning for non-destructive quality control, defect detection, mixing homogeneity assessment, and vitamin stability prediction. The second part analyzes methods for animal location and behavior detection. This article presents computer vision-based systems, including modifications of YOLO, for automatically tracking and classifying key behavioral patterns (lying down, standing, feeding, and aggression) in cattle and pigs, even in crowded conditions. It also discusses the use of ultra-wideband (UWB) systems and accelerometers combined with machine learning for high-precision positioning and detection of specific behavioral anomalies, such as lameness and playfulness. The third section focuses on the application of machine learning in veterinary diagnostics, including the automated interpretation of medical images (X-ray, ultrasound, and MRI) as sensor data streams for the diagnosis of cardiovascular, oncological, and orthopedic diseases in farm and small animals. Furthermore, the article examines the use of machine learning models for proactive disease diagnosis in farm animals and poultry based on multimodal data and image analysis. Considerable attention is given to methods and tools for radiometric diagnosis of animal diseases at an early stage using microwave sensors, as well as laser therapy and surgery in veterinary medicine. The review concludes that the integration of intelligent systems enables a transition to data-driven livestock management, significantly improving animal welfare and, consequently, the efficiency and sustainability of agricultural production.

## 1. Introduction

As the global population grows, the role of efficient livestock production as a key component of nutrition is growing. Current production technologies rely on manual observation and periodic data collection, are often subjective, labor-intensive, and lacking time sensitivity, leading to delays in identifying health or nutritional problems and, consequently, economic losses.

In response to these challenges, a rapid shift toward precision livestock farming (PLF) (part of digital agriculture) is underway. This concept involves the collection and analysis of large volumes of data in real time [1]. In this regard, the integration of intelligent technologies based on machine learning and neural networks with sensor systems based on intelligent sensors, IoT, and remote monitoring and control has become essential. These technologies enable objective and highly accurate monitoring of individual animals, enabling a shift from reactive to proactive management of production processes. Intelligent systems not only enhance operational efficiency but also play a central role in improving veterinary diagnostics, feed quality control, and understanding complex behavioral patterns. For example, quality control of feed additives, such as mineral and vitamin premixes, is crucial for animal health and productivity, and non-destructive testing methods based on sensors and microscopy provide the necessary accuracy. Accurate tracking of animal behavior, in turn, is one of the most sensitive indicators of their physiological state and stress level.

This review systematizes and analyzes recent advances in the application of intelligent technologies in three key interrelated aspects of animal husbandry:

(1) Quality assessment and optimization of mineral and vitamin premixes, including the use of microscopy for non-destructive testing, homogeneity assessment, and stability prediction using IR spectrometers, strain gauges, and machine vision sensors.
(2) Animal location identification and behavior analysis using computer vision (the YOLO neural network and its modifications), ultra-wideband (UWB), and inertial sensors (accelerometers) to detect anomalies such as lameness and aggression.
(3) Application of deep learning in veterinary diagnostics, including automated interpretation of medical images as data streams generated by digital sensors, proactive diagnosis of farm animal diseases based on multimodal data analysis, and the use of technical tools for early disease detection.

**Figure 1:**
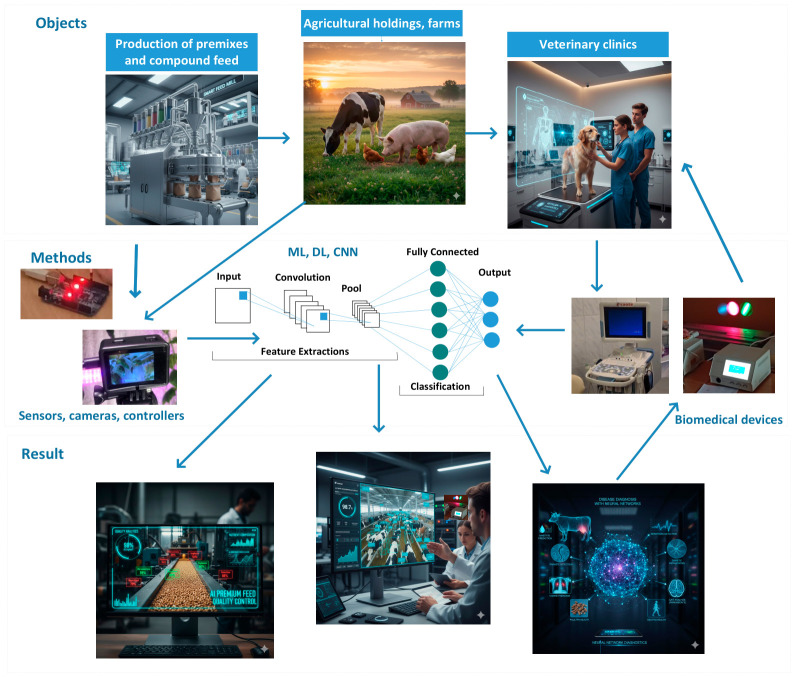
Structure of the review of intelligent methods and tools in animal husbandry and veterinary medicine (The figure was created by the authors; the images of the “Objects” and “Results” fragments were generated using AI; the “Methods” section uses photographs taken by the authors.).

To provide a systematic approach to digitalization in livestock farming, we proposed an integrated Feeding-Behavior-Health (FBH) model. Within this ecosystem, the three studied areas function as a closed-loop control system (Figure 1):

Input data control—monitoring the quality of premixes and feed using sensor data analysis determines productivity potential and basal metabolic rate.

Process monitoring—visual and sensory analysis of animal behavior in real time serves as an indicator of resource utilization efficiency and the impact of the external environment on the herd.

Veterinary diagnostics as a flow processing tool—data streams generated by digital sensors, requiring algorithmic interpretation, together with laser and radiometric therapy methods, complete the loop, enabling adjustments to animal health, which, in turn, requires adaptation of diet or housing conditions.

Despite a growing number of publications, existing reviews in the PLF field [2,3,4,5] often limit themselves to descriptive summaries of methods without systematic analysis. Readers find it difficult to assess the actual readiness of technologies for industrial implementation based solely on accuracy metrics. The contribution of this article is to propose a new analytical framework that compares AI methods and sensors along two dimensions: technology readiness level (TRL) and the quality of the presented evidence (Section 3.1, Section 3.2, Section 3.3 and Section 3.4). Our review is unique in that the strength of the evidence is methodologically assessed based on field and laboratory testing, dataset size, and external validation (Section 2).

This approach enables us to derive a new synthesis that: critically evaluates the robustness of technical areas of PLF (1); explicitly identifies gaps in the implementation of promising but unproven methods (Section 4.1) (2); and offers a list of recommendations for improving the robustness of future PLF research aimed at industrial implementation (Section 4.2) (3). This article is not only a literature review, but also a methodological tool for critically assessing the potential of various intelligent solutions in PLF.

The aim of this study is to comprehensively review and summarize methods for integrating smart sensors and deep learning algorithms that consider feed premix parameter control, ethological monitoring, and medical imaging processes as multimodal digital data streams for proactive management in precision livestock farming.

## 2. Materials and Methods

This systematic review was conducted in accordance with the Preferred Reporting Items for Systematic Reviews and Meta-Analyses (PRISMA) 2020 guideline [6], PRISMA 2020 Main Checklist can be found in Supplementary Materials (Table S1).

### 2.1. Topics and Geography of Literature Search for Review

**Figure 2:**
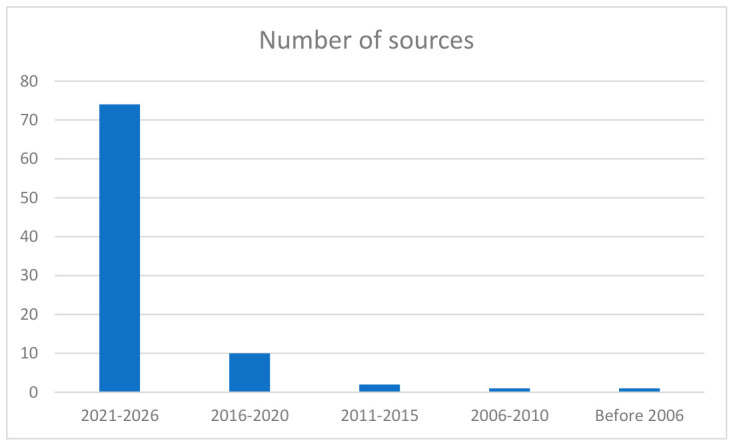
Literature review by publication period.

**Figure 3:**
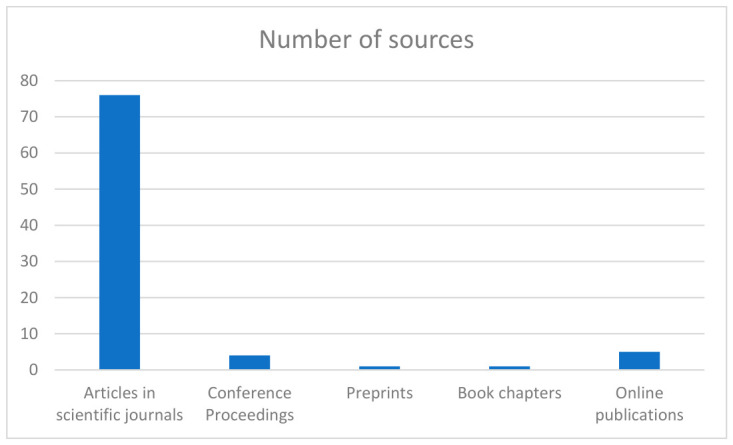
Literature review by publication type.

**Figure 4:**
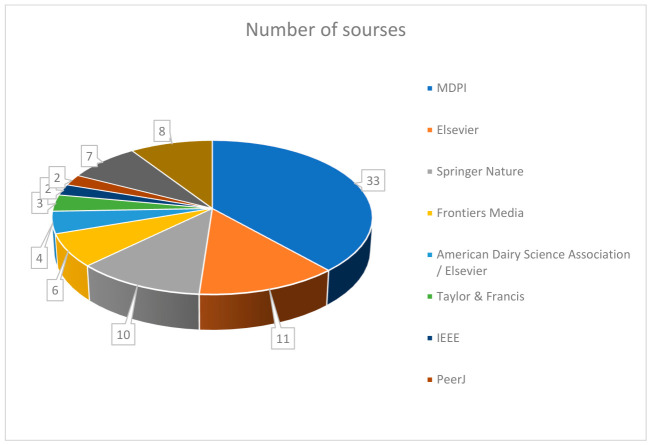
Distribution of Articles by Publisher.

This literature review includes articles and conference proceedings on animal husbandry, agricultural engineering, and biomedical engineering. Sources included publications in the Scopus and Web of Sciences scientometric databases, as well as Google Scolar. The review primarily focused on English-language literature, with some Ukrainian and Russian-language publications. The dominant publishers included MDPI, Frontiers, Taylor and Francis, JAMA Network, Science Direct, and the most well-known conference proceedings platforms, IEEE Xplore, CEUR-WS and IOP. The search focused primarily on recent literature written within the last five years (Figure 2), but earlier publications of interest were also included, particularly those related to the development of technical tools in biomedical engineering, which is a promising area, and feed requirements. The majority of the reviewed publications were published in scientific journals; conference proceedings, monograph chapters, preprints, and online publications were also included (Figure 3). The distribution by well-known scientific publishers, with journals indicated, is shown in the table; a visual summary diagram is shown in Figure 4. The distribution of articles by publishers and journals is shown in Table 1.

Table 2 provides an analysis of publications in this review by author affiliation (the first author or lead research team), indicating the research topic. Authors from China lead the number of publications in this topic, followed by scientists from Italy, the United States, India, and South Korea. Preprints and online publications are not included in this table. This selection focuses primarily on applied IT solutions (computer vision, object recognition), where Chinese universities currently lead, and on advanced veterinary visualization, where Italian schools (e.g., the University of Padova) are strong. Many articles in MDPI and Frontiers are the result of work by international teams, but the country of the main research center (Lead Affiliation) is included here.

**Figure 5:**
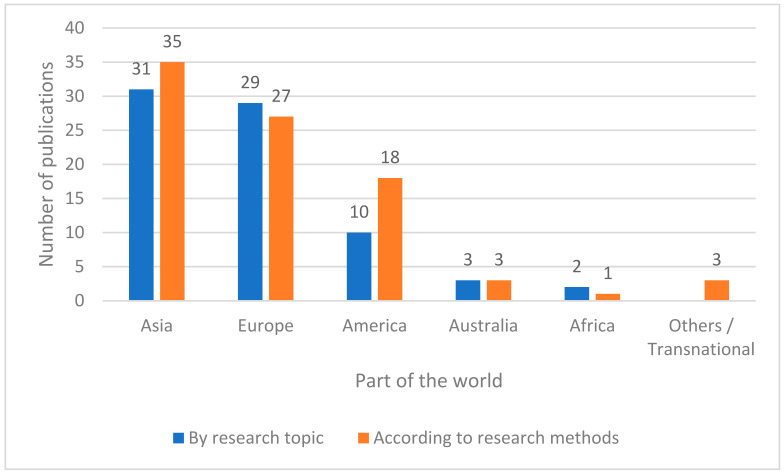
Distribution of review article topics and methods by continent.

Table 3 lists research centers by country (including preprints and online publications), indicating their research methods. Figure 5 summarizes the data in Table 3 and Table 4 by continent. Thus, we see that Asian researchers (primarily China) have produced the largest number of recent publications on this topic, followed by European researchers, and third place by scientists from the Americas.

The article includes three main chapters. The first section provides an overview of new technologies in the production of animal feed and premixes. The second section covers intelligent technologies for monitoring animal behavior. The third section is devoted to a review of machine learning methods, electronic devices for diagnosing animal diseases, and laser technologies for therapy and surgery in veterinary medicine.

### 2.2. Selection Criteria

**Figure 6:**
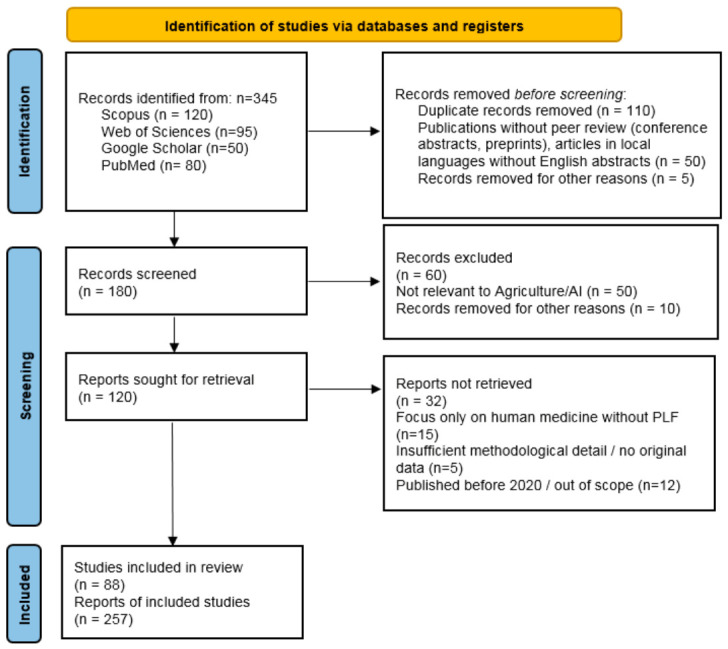
PRISMA flow diagram of the study selection process.

Studies based on traditional methods of manual observation and data collection (manual weighing, clinical examination by a veterinarian), simple automation systems without intelligent analysis (timed feeders), and classical statistical methods that do not utilize machine or deep learning algorithms were excluded from the analysis. Theoretical concepts of IoT systems without experimental testing on farm animals were also excluded. Therefore, the review focuses specifically on intelligent and biomedical engineering methods, rather than on automation in animal husbandry in general. The inclusion and exclusion criteria are presented in Table 4. The query is shown in Listing 1. The PRISMA flowchart, which illustrates the systematic process of publication selection for this review, is shown in Figure 6.

**Listing 1.** Literature search query for Scopus/Web of Science.

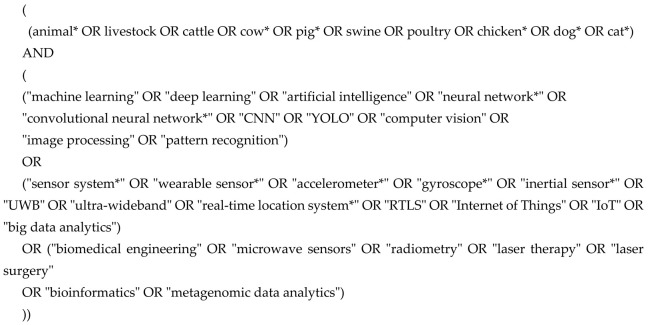



### 2.3. Quality Assessment of Evidence

To ensure the objectivity and scientific rigor of the systematic review, a critical assessment of the quality and strength of evidence presented in the selected original studies (*n* = 88) was conducted. The assessment was conducted not based on formal bibliometric metrics (citation rate, journal impact factor), but on the methodological characteristics of each study, which determine the reliability and generalizability of the results. Critical synthesis was conducted using three key criteria:

1. Trial conditions (test environment). Studies were classified by experimental design into “Field trials” (studies on real commercial farms under natural production conditions) and “Laboratory studies” (tests in strictly controlled vivarium conditions, experimental farms, or on small animal samples). Evidence obtained during long-term field trials was considered stronger and more representative for assessing the prospects for the real-world implementation of precision livestock farming technologies.
2. Data set size and representativeness. The volume of data used for training, validating, and testing the artificial intelligence models (number of animals, images, hours of video, or sensor recordings) was analyzed. Studies with large, heterogeneous, and representative datasets (e.g., >10,000 images or >100 animals) collected under diverse conditions were considered more reliable and less susceptible to overfitting.
3. Availability of external validation. The presence of a hold-out set/fertile set validation of the trained model was critically assessed. The presence of external validation was considered key confirmation of the high generalization ability of the model and its readiness to operate under unpredictable real-world production conditions.

The results of this critical assessment were integrated into the technical sections of the review (Section 3.1, Section 3.2 and Section 3.3) and used for a comparative analysis of the technology readiness levels (TRLs) of various intelligent methods in animal husbandry (Section 4).

### 2.4. Data Synthesis and Analysis

Due to the high methodological heterogeneity of the included studies—which utilized distinct sensor types, diverse AI architectures, and non-combinable outcomes—a quantitative meta-analysis was not feasible; thus, a qualitative narrative synthesis was conducted. Studies were grouped into synthesis categories based on the Feeding-Behavior-Health (FBH) framework and evaluated for suitability based on dataset scale, test environment (field vs. lab), and external validation. Data preparation involved extracting and tabulating standardized performance metrics (Accuracy, Precision, mAP, MAPE) across individual studies without statistical transformations. Synthesis outcomes, along with Technology Readiness Levels (TRLs) and geographic distribution, were visually structured using systematic summary matrices (Table 1, Table 2, Table 3 and Table 4, Figure 2, Figure 3, Figure 4 and Figure 5). Potential causes of heterogeneity were explored through subgroup analyses based on TRLs, publication timeline, and geographic leadership. Finally, a sensitivity analysis was performed by cross-examining the robustness of the core conclusions when omitting lower-quality evidence that lacked independent validation or field testing.

### 2.5. Quality Assessment and Risk of Bias

The methodological quality and risk of bias of the included studies were assessed independently by two authors (N.K. and M.P. for Section 3.1, N.K. and D.H. for Section 3.2, L.S. and T.H. for Section 3.3), and any disagreements were resolved by consensus or discussion with a third author (Yu.M., Ie.A., and P.K. for Section 3.1, Section 3.2, and Section 3.3, respectively). Because this review focused on technological and algorithmic applications rather than clinical trials, a structured quality assessment system adapted for research in the field of machine learning and sensors was applied. Articles were critically assessed across four key areas: representativeness and scale of the dataset, clarity of sensor and hardware specifications, robustness of model validation (internal cross-validation versus external field testing), and completeness of reporting.

The risk of bias and methodological quality of the included studies were evaluated independently by two reviewers based on four technical domains: dataset scale, hardware specification clarity, model validation robustness, and reporting completeness. Discrepancies were resolved through consensus, and no automation tools were used.

The primary outcomes and performance measures extracted from the evaluated studies include classification and detection metrics (Accuracy, Precision, Recall, F1-score, and mean Average Precision [mAP]), as well as regression and prediction errors (R^2^, RMSE, and MAPE). No clinical effect measures, such as risk ratios or mean differences, were applicable due to the technical nature of the synthesis.

Reporting bias was assessed by comparing the published methodology of each study against its reported outcomes to ensure all evaluated models and sensor tests were fully disclosed. Potential publication bias was critically evaluated by checking for the underreporting of negative results, such as low algorithmic accuracy or hardware deployment failures.

The certainty of the overall evidence was assessed qualitatively based on the maturity and validation level of the analyzed technologies. Higher confidence was assigned to findings backed by high Technology Readiness Levels (TRLs), large-scale datasets, and independent external validation, while laboratory-only setups without field testing were downgraded in certainty.

## 3. Results

### 3.1. Optical and Analytical Sensor Systems for Quality Control of Mineral and Vitamin Premixes

#### 3.1.1. Evaluation of the Composition and Stability of Mineral and Vitamin Premixes

Balanced mineral and vitamin complexes are key factors in regulating metabolism, ensuring optimal growth dynamics, and maintaining high reproductive potential in livestock (Byrne & Murphy) [7]. The effects of the components on animal vital functions are presented in Table 5.

Premix composition requires precise dosing of micronutrients such as zinc, copper, manganese, and iron, as well as fat-soluble vitamins A, D, and E, which are active substances in many physiological processes. Recent studies have shown that the bioavailability and chemical form of these micronutrients are critical factors influencing animal productivity and environmental safety (Yang et al.) [8].

**Table 5:** Effect of a feed component or premix on animal vital functions.

Animals	Feed or Premix Components	Impact Indicator	Source
All animals	Zinc, copper, manganese, and iron	Productivity, Environmental Safety	[9]
Dairy cows	Mineral and vitamin component ratios	Milk Production and Blood Biochemistry	[10]
Boiler chickens	Sodium chloride levels	Digestive Enzyme Activity and Osmotic Balance	[11]
Birds	Sodium chloride concentration	Intestinal Morphology	[12]
Dairy cows	Sodium chloride concentration	Osmotic Stress and Changes in Gut Microbiota Composition	[13]
Tropical ruminants	Use of organic chelates	Mineral Retention in Animal Tissue, Reduction in Excretory Losses, and Reduction in Environmental Pollution	[14]
All animals	Replacement of inorganic elements with organic ones	Animal Growth	[15]
All animals	Moisture, shelf life, and particle size distribution	Component Stability, Premix Bioefficiency	[16]
All animals	Heat recovery, optimized electric drives, and process planning	Reduction in Energy Consumption	[17]
All animals	Digital monitoring tools and predictive maintenance systems	Minimization of Production Losses	[18]
All animals	Raw material dosing accuracy and mixing uniformity	Final Mix Coefficient of Variation	[19]

According to Syrovatko’s findings [9], qualitative optimization of diets correlates with increased milk yield and improved blood biochemistry in cattle. The author notes that a deficiency of trace elements such as zinc and copper is a key factor in weakened reproductive health and the increase in pathologies. Zhang et al. [10] also found that a carefully calibrated sodium chloride content in premixes stimulates digestive enzymes and maintains osmotic balance in broilers, ultimately increasing the feed conversion ratio.

Additional research by Chen et al. [11] identified a relationship between chloride concentration and intestinal morphology in poultry, demonstrating that controlled sodium and chloride intake promotes intestinal villus development and improves nutrient absorption. In contrast, excess NaCl levels have been associated with osmotic stress and altered gut microbiota composition (Speck, Bannink, & Dijkstra) [12].

According to research by McDowell and Arthington [13], the use of organic chelating compounds promotes more efficient mineral accumulation in tissues, reducing their excretion from the body and lowering environmental impacts. These findings are supported by the work of Xiong et al. [14], who demonstrated that the use of organic micronutrients, even at reduced dosages, allows for the maintenance of livestock growth rates. This approach offers an environmentally sound alternative to inorganic salts within the framework of sustainable feed production. It is worth noting that the stability of premix ingredients is largely determined by external factors: humidity levels, shelf life, and particle size distribution.

In general, the degree of stability of premix components depends to varying degrees on environmental conditions such as humidity, storage duration, and particle size distribution. Moisture absorption can lead to aggregation and oxidative degradation of vitamins, especially A and E (Ye et al.) [15]. Therefore, maintaining the recommended microclimatic parameters during storage is crucial to ensure consistent quality and bioefficiency of premixes.

#### 3.1.2. Artificial Intelligence for Processing Sensor Data Streams in Quality Control of Compound Feed and Premixes

The production of mineral and vitamin premixes is a complex technological process involving precise dosing, uniform mixing, dust protection, and continuous quality control. Modern feed mills are increasingly implementing energy-efficient and automated solutions to improve production stability and sustainability (Bayan et al.) [16]. Industrial energy audits have shown that feed and additive manufacturing facilities can reduce overall energy consumption by up to 25% through the use of efficient heat recovery systems, optimized electric drives, and advanced process planning.

Sustainable energy management is becoming an integral part of the feed milling industry’s modernization strategy. Liakos et al. [17] emphasize that the implementation of digital monitoring tools and predictive maintenance systems can significantly minimize production losses and improve the environmental impact of agri-food production. In modern premix plants, energy-intensive components such as mixers, pneumatic conveyors, and dust filters are increasingly controlled by integrated control systems that continuously evaluate operating efficiency and energy consumption. Process reliability and stability largely depend on the accuracy of raw material dosing and mixing uniformity. Pasha et al. [18] simulated the process of combining powdered feed components, establishing a direct relationship between mixture quality and particle size distribution and moisture content. The study confirmed that the implementation of digital twin technology allows for the effective simulation of process dynamics and the determination of the ideal mixing duration. This not only reduces energy costs but also minimizes compositional deviations between different production batches.

Developing the topic of automation, Gao et al. [19] presented a feed preparation system using high-precision strain gauge dosing and sensor feedback. This solution achieved a 98% homogeneity level of ingredients, demonstrating that automation is the only option for consistent quality in large-scale enterprises (Figure 2).

At the same time, material quality control methods are being improved. You et al. [20] described a sensor system for the rapid monitoring of contaminants and moisture deviations directly during the production process. This technology is based on the integration of infrared and capacitive sensors with microcontroller control, which ensures an instant response to changes in parameters.

#### 3.1.3. Processing Data Streams from Machine Vision Sensors in Quality Control of Compound Feed and Premixes

Advances in artificial intelligence, particularly machine learning and computer vision, have radically transformed the agricultural sector. The implementation of intelligent technologies has enabled unprecedented levels of automation and precision in product quality control. In the context of the digitalization of agricultural supply chains, the integration of deep learning algorithms, edge computing concepts, and next-generation sensor systems enables direct monitoring of feed and premix characteristics during the production process. A summary of these methods is presented in Table 6.

Liakos et al. (2018) [21] presented one of the first comprehensive reviews on the implementation of machine learning (ML) in agriculture, highlighting supervised and unsupervised algorithms that facilitate yield forecasting, defect detection, and image-based classification of agricultural inputs. Their analysis confirmed that neural network-based methods outperform traditional regression and clustering methods when dealing with complex, heterogeneous agricultural data.

**Table 6:** Machine learning methods and technical means for visual determination of the quality of compound feeds and premixes.

Method	Result	Source
Digital twins, strain gauges, and feedback	Modeling mixing dynamics and predicting optimal mixing times, reducing energy consumption	[19]
Sensor system based on IR and capacitive sensors	Detecting particle contamination and moisture anomalies during feed processing	[20]
ResNet and EfficientNet	Detecting surface defects and texture anomalies in processed materials (powders and grains)	[22]
Edge-AI (lightweight CNN models directly on embedded devices near production lines)	Achieving real-time defect detection with minimal latency directly on the line	[23]
ML and hybrid control systems	Optimizing feed formulations, reducing waste	[24]
DL for texture and color analysis	Identifying impurities and assessing particle size variations in premixes.	[25]
Image preprocessing pipelines (segmentation and contrast)	Improving image classification accuracy for visually assessing premix samples under varying lighting and humidity conditions	[26]
Data augmentation and generative adversarial networks (GANs)	Creating synthetic training data, enabling the recognition of small defects such as salt clumping or microcontamination.	[27]
CNN, Vision Transformers	Classifying contamination levels in powdered products	[28]
DL on RGB images	Detecting foreign particles in feed	[29]
Textural feature extraction using DL	Assessing particle homogeneity is an indicator of the proper distribution of ingredients in premixes. Automatic adjustment of mixing times and batch rejection criteria based on AI video data.	[30]
Closed-loop systems (visual sensors, CNN, PLC, SCADA)	Gut microbiota and its correlation with clinical health indicators (growth, feed conversion)	[31]
ML analysis of metagenomic sequencing data	Concentration of key vitamins in premix	[32]
PLS, NIR	Mixing quality based on visual particle distribution patterns	[33]
Image processing and ML	Detection of adulteration and confirmation of the authenticity of high-value feed additives	[8]
SVM, random forest + Raman spectroscopy	Prediction of the stability and degradation rate of vitamins (C, B1, etc.) in premixes depending on temperature, humidity, and storage time	[34]
ANN	Prediction of egg production costs based on nutrient requirements	[35]
Cascaded ANN with softmax function	Measurement of trace elements and mineral content in mammalian samples	[36]
ICP-MS (mass spectrometry)	Result	[37]

Ding et al. [22] analyzed the effectiveness of deep learning (DL) methods for quality assurance in the food industry. The researchers demonstrated that convolutional neural networks (CNNs), in particular the ResNet and EfficientNet architectures, can accurately identify surface defects and structural anomalies in bulk raw materials, including grains and powdered ingredients. These methods provide a methodological basis for quality control of feeds where visual heterogeneity of salt and mineral components makes manual control difficult.

According to [23], the Edge-AI concept involves deploying optimized (lightweight) CNN models directly on edge devices installed along production lines. This approach enables real-time defect detection with extremely low latency. For premix plants, the use of local computing is critical, as any delays in data transmission to the cloud can lead to delayed or erroneous product quality assessments.

From a systems integration perspective, Basu and Narayan [24] examined how machine learning pipelines facilitate the broader digital transformation of agricultural practices. They emphasized hybrid decision-support systems that integrate sensor data, historical production logs, and visual inspection results to optimize feed formulations and reduce waste. Cao, Sun, and Bao [25] provided a comprehensive review of computer vision applications in crop management, focusing on deep learning models capable of recognizing texture, color, and edges. While these same analytical methods focused on plant phenotyping tasks, they can be transferred to premix quality assessment, particularly for identifying impurities and granularity variations.

An additional study by Suda [26] analyzed multiple computer vision and machine learning systems in the food processing sector and proposed preprocessing pipelines—illumination normalization, contrast enhancement, segmentation, and feature extraction—that significantly improve image classification accuracy. These procedures are directly applicable to the visual assessment of premix samples under varying illumination and humidity conditions, where uneven illumination or condensation can distort pixel characteristics.

A key obstacle to developing deep learning models for feed monitoring is the lack of large and labeled datasets. Shorten and Khoshgoftar [27] systematized image augmentation methods, including geometric transformations, noise superposition, and the use of generative adversarial networks (GANs) to synthesize new samples. These approaches allow for the artificial increase of training samples and the prevention of overfitting. This is necessary for building highly accurate CNN models capable of detecting critical anomalies such as clumping or the presence of microparticles of contaminants in salt mixtures.

In [28], AI-based visual inspection methods were applied to food processing lines using CNNs and Vision Transformers to classify contamination levels of powdered products. Their approach achieved classification accuracy exceeding 97%, demonstrating that similar architectures can effectively distinguish between acceptable and defective premix batches. In a similar study, Chuang et al. [29] developed a deep learning-based inspection system for foreign particle detection in animal feed, confirming that convolutional neural networks trained on RGB images outperformed a traditional threshold-based method by a factor of 2.5 in accuracy and recall. Anukiruthika et al. [30] extended this paradigm to granular agricultural materials, showing that textural feature extraction using convolutional neural networks can be used to assess particle uniformity, a critical indicator of proper ingredient distribution in mineral-vitamin premixes. Agrawal et al. [31] examined the integration of computer vision modules with industrial automated systems in feed production. The paper presents a closed-loop control architecture in which visual sensors provide feedback to PLCs and SCADA systems. This enables automatic adjustment of process parameters, including mixing duration and product rejection conditions. This synergy between AI and automation is the foundation for the transition to fully autonomous quality management in the industry.

A literature review shows that these supplements stabilize the gut microbiome, improve digestion, and boost immunity. Machine learning is often used in studies to analyze complex gut microbiota sequencing data and correlate it with clinical health indicators (growth, feed conversion) [32]. The use of partial least squares for processing NIR spectrometer data transforms complex chemical analysis into an instant procedure. Now, the presence of key vitamins in feed mixtures can be assessed without laboratory reagents and raw material loss [33].

The use of various image processing methods and machine learning to quantitatively assess the uniformity of micronutrient distribution in premixes. Models classify mixing quality based on visual patterns of particle distribution [8]. The use of portable Raman spectroscopy and machine learning classifiers (e.g., SVM, random forest) for rapid detection of adulteration and confirmation of the authenticity of high-value feed additives included in premixes [34].

Development of artificial neural networks (ANN) to predict the stability and degradation rate of vitamins (C, B1, etc.) in premixes depending on temperature, humidity, and storage time [35]. Multivariate polynomial models and ANNs were tested to predict egg production costs based on essential amino acid consumption and time. A cascaded ANN with a softmax activation function demonstrated the best results. The optimal economic scenario ($0.873/kg egg) after 20 weeks was achieved with a daily consumption per head of 943.7 mg lysine, 858.3 mg methionine + cystine, and 876.8 mg threonine. The use of ANNs in poultry farming allows for more accurate ration calculations, increasing enterprise profitability [36].

The importance of micronutrient balance for healthy organisms is also emphasized, as their deficiency or excess provokes severe pathologies (diabetes, cardiovascular, and neurological diseases). Inductively coupled plasma mass spectrometry (ICP-MS) is proposed as a reference method for analyzing mammalian tissues and serum. The study confirmed that maximum accuracy (minimum RSD deviation) is achieved with microwave digestion of samples in high-purity nitric acid. This protocol simplifies and accelerates precision monitoring of mineral composition in veterinary and human medicine [37].

Thus, sources [19,20,31] show the complete chain from a sensor on a conveyor to a control command to stop the line. A group of sources [22,23,24,25,26,27,28,29,30] demonstrates all stages of working with visual data: how to prepare a photo, how to train a neural network, and how to run it directly at the factory (Edge AI). Sources [33,34] highlight methods of authenticity and chemical control that replace time-consuming laboratory tests.

To summarize, this section compares traditional control methods with modern intelligent systems aimed at dosing accuracy and component stability. AI and computer vision technologies used in feed and premix production can be used in the following ways:

(1) CNN—ResNet and EfficientNet architectures are used to detect surface defects and texture anomalies in powders and grains.
  - Edge-AI—deploying models directly in production allows for real-time defect detection with minimal latency;
  - GANs are used to create synthetic data, which helps neural networks recognize rare defects, such as clumping.
(2) Sensory and spectroscopic classification methods are used in feed production in the following ways:
  - NIR spectroscopy + ML allows for rapid and non-destructive sample assessment of key vitamin concentrations.
  - Raman spectroscopy is used to confirm the authenticity of high-value additives and detect counterfeit products.
  - Digital twins simulate the mixing process, helping to find optimal timing and reduce plant energy consumption.

A critical synthesis of non-destructive quality control methods for feed raw materials and premixes reveals significant differences between NIR and Raman spectroscopy in terms of their applicability in production settings. NIR-based systems using partial least squares (PLS) [17] or deep learning [28] algorithms demonstrate high accuracy in the rapid analysis of the concentration of macronutrients and key vitamins (e.g., vitamin A [15]). The main advantages of NIR are the relatively low cost of equipment and high scanning speed. However, this method has a significant drawback: the high complexity of model calibration, which requires the creation of extensive and heterogeneous training datasets that take into account the variability of raw material batches, temperature, and humidity [27,28]. Unlike NIR, Raman spectroscopy methods combined with machine learning classifiers (SVM, Random Forest) [21,26,28,34] demonstrate higher specificity in identifying specific chemical bonds. This makes Raman spectroscopy the preferred method for rapid detection of adulteration and confirmation of the authenticity of high-value feed additives and amino acids included in premixes [22,27]. The main disadvantage of Raman spectroscopy remains the higher cost of equipment and sensitivity to sample fluorescence, which may limit its use for analyzing complex, heterogeneous mixtures.

Thus, the choice of non-destructive testing method depends on the production task: for routine monitoring of mixing homogeneity and concentration of essential nutrients, optimized NIR systems are more effective [17,21]. At the same time, to control the authenticity of raw materials and protect against counterfeiting at the production input, it is advisable to implement systems based on Raman spectroscopy [21,26,34]. The Internet of Things technology in the production of premixes is described in the work [38].

A critical comparison of intelligent methods for quality control of feed and premixes is provided in Table A1.

Overall, artificial intelligence and computer vision technologies are changing the approach to feed quality assessment, transforming a labor-intensive process into a data-driven paradigm. Deep learning architectures, supported by robust data augmentation strategies and edge computing hardware, enable the accurate detection of physical and compositional anomalies in premixes. Integrating these tools into automated production environments ensures not only consistent product quality but also improved traceability and operational efficiency—key prerequisites for sustainable and intelligent feed production.

### 3.2. Determining the Location and Behavior of Animals Using Machine Learning

With the growing demand for efficient, sustainable, and humane livestock production, it is crucial to continuously and accurately monitor the well-being and comfort of farm animals. Animal welfare, early disease detection, and optimized resource management (feed, pasture) directly depend on the ability to track their location and behavior patterns (Table 7).

Traditional monitoring methods based on manual patrols and visual observation are subjective, inconsistent, and insufficient to cover large herds or farms. This leads to late detection of problems, reduced productivity, and increased operating costs. This chapter examines the area where machine learning is becoming a key technological enabler for the transformation of livestock management. The use of machine learning algorithms enables the automated real-time analysis of huge amounts of data coming from various types of sensors, such as GPS trackers, accelerometers, cameras, and microphones, installed on animals or in their environment. Yu et al. [38] filmed a video in a real barn (not a laboratory), where many cows overlapped each other. The authors take YOLOv5 and add a combination of Res-Net and DensNet (Res-Dense) to enable the model to better visualize the animal in shadows and with partial occlusion. The researchers classify typical everyday activities—lying down, standing, feeding, drinking—and demonstrate that, with the right architecture, video can replace wearable sensors for behavioral analysis. This directly qualifies as an example of “vision-based everyday behavior.”

The paper by Li et al. [39] discusses a new dataset for video-based cow behavior recognition (CBVD-5). The value of the paper lies not in the model, but in the dataset: they collected videos in a very dense barn (~80% occupancy), where cows often merge into a single “mass.” The researchers identified five key behavioral responses necessary for welfare monitoring: standing, lying down, eating, drinking, and chewing. Basic deep learning models are shown, and accuracy is reduced precisely by crowding, meaning this is a real-world, not a “pure,” problem. The study is the source of a real-world video dataset of indoor cattle behavior.

Han et al. [40] track cattle in a crowded barn using deep learning. The paper describes not only frame classification but also linking the same cow across frames (multi-object tracking). Problem: In a barn, cows walk very close to each other, frequently changing positions and partially obscuring the camera. The authors combine a detector and a tracker to store the ID of each animal for a longer period of time. This is necessary for subsequent calculations of “how long a specific cow was at the feeder/in the stall.” The study is a good example of “behavior tracking.” Wu et al. [41] tracked the respiratory behavior of cows using computer vision and deep learning. The level of complexity is higher: they do not simply look at a standing cow, but isolate its ribcage and count respiratory cycles. This is done for several cows in the frame simultaneously, making the system scalable to groups. Respiration parameters are then associated with the animal’s condition (stress, heat). This demonstrates that computer vision in animal husbandry is already moving towards “physiologically significant” parameters, and not just “standing/lying down.”

Li et al. [42] presented a modified RFR-YOLO neural network architecture optimized for identifying dairy cattle behavior patterns. A key innovation was the integration of spatial feature-extraction (RFR) units, which allowed the system to operate effectively in real farm conditions with noisy backgrounds. The study confirmed the model’s advantage in recognizing visually similar actions, such as chewing and passive standing. The authors highlight the contactless nature of the method as an important operational advantage: video analytics eliminates the need for maintenance and battery replacement in body sensors and collars. In turn, Che et al. [43] proposed an alternative approach to video stream analysis, using an improved FlowEQ-Transform neural network model for classifying cattle actions. Moravcikova et al. validated an ultra-wideband (UWB) positioning system for accurate cow tracking in a commercial barn [44]. The authors actually deployed UWB (Ultra-Wide Band) positioning in a working free-stall barn and measured the system’s errors when cows were moving/standing. It was shown that for tasks such as “was in the milking/feeding/resting zone,” the accuracy is sufficient, but not for very small movements. The influence of metal structures and antenna placement is discussed. A good “bridge” between the world of computer vision and the world of RTLS (Real-Time Location Systems).

A validation of a real-time localization system for indoor dairy cows (RTLS) is shown in the paper by Jocelyn et al. [45]. The focus is on the measurement methodology: how to correctly place control points and how to account for antenna height. The authors show the error spread and explain which areas of the barn are “worse.” Thus, this model can be used to analyze the presence and spatial patterns of a herd. This can be useful if the task is to recognize a location without video.

In the work of Adriaens et al. [46], a method for identifying moments of rest (lying down) of cows based on noisy 3D data of ultra-wideband (UWB) localization is presented. A distinctive feature of the study is the use of intentionally distorted input signals. The combined smoothing and classification algorithm developed by the authors allows for the successful interpretation of the movement trajectory, proving that even with low positioning accuracy, reliable data on animal behavior can be obtained. This is a clear example of effective noise filtering in localization systems. In turn, Vazquez-Diosdado et al. [47] demonstrated the possibility of quantitatively assessing the play activity of calves using similar location sensors. Sensor systems for classifying the behavior of dairy cows on pasture are presented in the article by Pichlbauer et al. [48].

**Table 7:** Application of machine learning models to assess animal behavior.

Biological Object	Method/Model	What Is Being Studied/Result	Source Number
Cattle	YOLOv5, RFR-YOLO, ResNet, DenseNet	Automatic detection of cow body position and actions (standing, lying, feeding).	[38,39,42]
Cattle (dairy herd)	Deep learning, RTLS, UWB (Ultra-Wide Band)	Tracking time spent near stalls, in the milking area, or in the resting area; high-precision positioning in the barn.	[40,44]
Cattle	Computer vision, FlowEQ-Transform	Assessment of physiological parameters (respiration, heat stress) and motion analysis using optical flow.	[44,46]
Cattle (herd)	RTLS, SEWIO, 3D-UWB with smoothing	Analysis of spatial patterns of herd behavior without the use of video, overcoming interference from metal structures.	[45,46]
Calves	Location and motion sensors	Assessment of play activity as an indicator of well-being and social development.	[47]
Cattle (on pasture)	Activity sensors, accelerometers	Monitoring of pasture behavior (rumination, body position, lameness).	[48,49]
Cattle/Pigs	Deep learning, CNN, LSTM	Comprehensive gait analysis for the early detection of lameness and specific movements.	[50,51,52,53,54,55,56]
Pigs	Deep learning	Classification of postures (lying, standing, sitting) as an indicator of comfort and health.	[57]
Pigs (in a group)	YOLOv7 + DeepSORT (tracking)	Individual tracking of trajectories in a group; detection of abnormal behavior as an early sign of illness.	[58,59]
Pigs	Machine vision (RGB cameras)	Early diagnosis of diseases based on changes in posture and movement dynamics.	[60]
Cattle, Pigs, Poultry	Deep learning (chain overview)	Detection, video tracking, and behavior classification in complex environments (occlusion, heterogeneous background).	[61]

**Figure 7:**
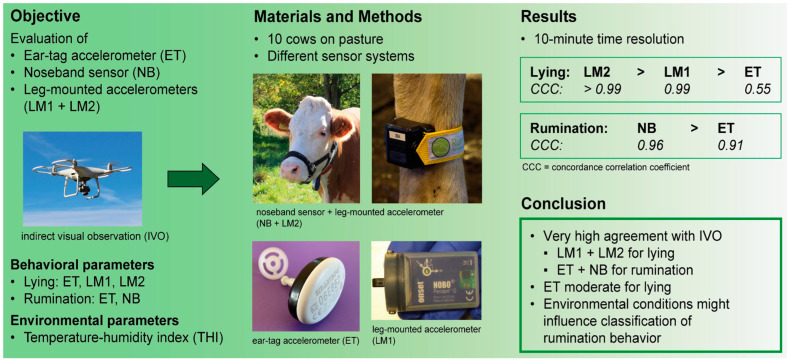
Sensor systems for classifying the behavior of dairy cows on pasture [49].

The authors compare several types of sensors (rumination, activity, body position) on pasture, where cows move more freely than in a barn. The researchers consider how often data should be sampled to maintain accuracy and show that some less expensive sensors also produce acceptable results (Figure 7) [49].

A study by Arabloui et al. [49] describes a method for identifying cattle activity based on accelerometer signals. The authors employ a standard analytical workflow: collecting data from a sensor attached to the animal, preprocessing the signal, extracting significant features, and then classifying them using support vector machines (SVMs) or random forests. The paper provides a detailed analysis of the dependence of recognition accuracy on the duration of time intervals (windows) and the complexity of the set of tracked actions. In turn, Mutawakil et al. [50] proposed using deep learning architectures for automated classification of cattle behavior based on accelerometer data.

Instead of manually inputting features, the researchers directly feed the raw signal into a CNN/LSTM, demonstrating that deep learning is better at capturing the differences between “standing chewing” and “just standing.” The authors also discuss the problem of unbalanced classes (some behavioral responses are rare).

The study by Pongsanun et al. [51] examines the classification of individual cow behavior based on accelerometry, gyroscopy, and a combination of both. Experiments have confirmed that while an accelerometer can handle basic tasks, integrating a gyroscope significantly improves the accuracy of identifying specific animal postures. The study is particularly valuable due to the length of the observation period (90 days), which closely approximates experimental conditions to real production processes. The authors also conducted a comparative analysis of the effectiveness of single sensors and integrated sensor systems.

Intelligent sensing and early warning for livestock based on wearable sensors is demonstrated by Ding et al. [52]. This is a review of the types of wearable sensors already in use for cattle, sheep, and pigs (IMUs, temperature, GPS/UWB, biosensors). The authors explain how these signals are used to detect behavior and build “early warnings” (e.g., disease or calving) based on them, and also consider the issues of power consumption and portability.

Yu et al. [53] developed an intelligent wearable device for real-time monitoring of livestock. This is an engineering work: they designed a special collar/device that collects activity parameters and physiological parameters in real time. The paper describes the architecture, power source, and data transmission. The authors show the results of animal testing and that the data is suitable for further processing using machine learning. 

**Figure 8:**
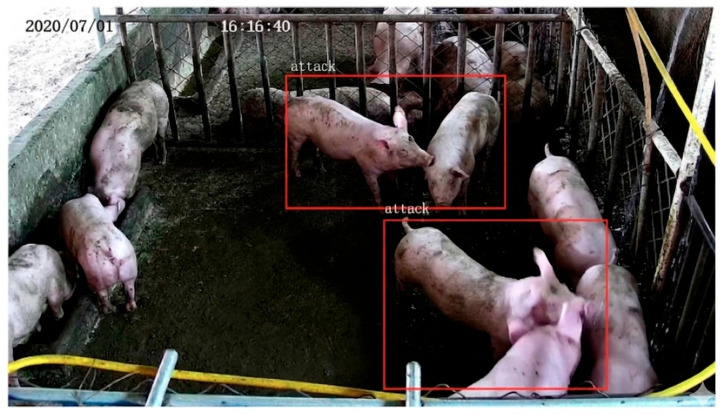
An analysis of the effectiveness of computer vision systems for detecting agonistic (conflict) behavior in pigs directly in feeding areas [55].

The work by Khan et al. [54] presents an analysis of the effectiveness of computer vision systems in identifying aggressive interactions between pigs in feeding areas. The study focuses on the automation of monitoring the conflict behavior of animals at feeding troughs. A camera above the feeder and computer vision distinguish normal pig behavior from aggressive or competitive behavior (pushing, crowding). The authors justify the need for this, since aggression affects stress and growth, and compare automatic detection with human marking. This is an example of how behavior directly affects welfare. Deep learning-based detection of aggressive behavior in pigs using an improved version of YOLO.X is shown in the paper by Li et al. [55]. The researchers take attack episodes (biting, chasing) and train an improved version of YOLO.X with an attention mechanism to avoid losing objects during overlaps. This provides high accuracy in a farm setting. The authors emphasize that this may be due to the fact that, with an automatic alert system, this is a good example of a deep learning detector with non-standard behavior (Figure 8).

In parallel, Zhang [56] describes a method for object recognition and animal condition monitoring based on deep learning algorithms. The proposed approach involves two-stage processing: the first stage detects pigs in the frame, and the second classifies their current state (rest, aggression, or active movement).

Pose classification of pigs using deep learning is described in the paper by Jeon et al. [57]. The goal is to consistently detect pig posture (lying, standing, sitting), as this is a key indicator of health and comfort. Research shows that deep learning maintains stable accuracy even with changing lighting. It is also proposed to use it in microclimate and feeding control systems. This is a crucial building block for larger behavioral systems.

Machine learning-based activity tracking of individual pigs was described by Deutsch et al. [58]. The focus is on individual tracking within a group: the trajectory of a specific pig is isolated, and based on this, activity is classified (lying down, walking, or active). Research shows an accuracy of around 90% even without expensive sensors, emphasizing that this can be done in near real-time.

Anomalies in pig health based on behavioral patterns during periods of activity were detected using deep learning by Tran and Thanh [59]. The idea behind the study is that if a pig behaves differently from others during the same period of activity, this may be an early sign of disease. The authors use YOLOv7+ tracking (similar to DeepSORT) to initially extract behavioral patterns and then search for deviations.

A study by Nasim et al. [60] presents a method for early disease diagnosis in pigs using machine vision systems based on RGB cameras. The authors analyze a set of visual markers, including changes in posture, movement dynamics, and specific behavioral signals. This approach enables the identification of pathological conditions at early stages using standard video recording equipment.

Deep learning for visual monitoring of animals, including detection, tracking, and behavioral analysis, is demonstrated by Rajagukguk et al. [61]. This is an overview of the entire chain: animal detection, video tracking, and behavior classification. The study covers the monitoring of cattle, pigs, and poultry, with a special focus on the advantages of deep learning architectures over traditional algorithms (in particular, their robustness to complex backgrounds). A separate block of work is devoted to the operation of multi-camera configurations and methods for overcoming the occlusion problem (object overlap). In addition, D’Urso et al. consider the implementation of real-time positioning systems (RTLS) for continuous monitoring of a dairy herd [62]. The authors show an example of the use of the SEWIO system: how it was installed in a barn, what the actual accuracy is in different zones, and how to conclude from these coordinates that an animal was “in the feeding/lying zone.” The researchers note the presence of certain technical barriers, such as the influence of metal structures, the occurrence of reflected signals, and the emergence of blind spots. However, the key conclusion of the study is the confirmation of the effectiveness of real-time positioning systems (RTLS) for analyzing animal behavior, provided that the data is properly processed.

This chapter is devoted to the transformation of traditional visual inspection methods into automated systems for monitoring physiological status and early diagnostics of pathologies.

For cattle, the primary focus is on high-precision positioning (UWB, RTLS) and determining physiological status (respiration, lameness, stress) through video analytics (YOLO, CNN). For pigs, the study focuses on individual tracking within a group and posture analysis. Behavioral deviations from group patterns are a marker of disease onset. For poultry, deep learning technologies are used for general activity monitoring and robust object recognition against a complex background. Comparison of computer vision methods and UWB sensors for various criteria for animal monitoring is presented in Table 8.

A comparison of AI methods for animal behavior recognition is shown in Table A2.

This section examines the transition from manual surveillance to automated well-being monitoring and early disease detection systems. Two components can be identified here.

(1) Visual monitoring or computer vision:
  - The YOLOv5 model and its modifications (with Res-Dense units, etc.) allow tracking cows and pigs even in dense crowding and shadows.
  - DeepSORT is used for continuous tracking of a specific animal’s trajectory in a herd.
(2) Sensor systems and wearables:
  - UWB (ultra-wideband) provides high-precision indoor positioning, helping to analyze the time an animal spends at a feeder or resting area.
  - Accelerometers and gyroscopes collect movement data (chewing, activity), which is then classified by algorithms (SVM or Random Forest) to identify abnormalities such as lameness.

### 3.3. Deep Learning for Multimodal Data Processing from Medical Imaging Sensors

Machine learning has found widespread application in both human and veterinary medicine. Deep learning methods have revolutionized the interpretation of complex medical signals and images, including in the diagnosis and treatment of heart, eye, skin, and tumor diseases.

Emphasize the role of the “sensor” in medical imaging: in veterinary diagnostics (X-ray, MRI, ultrasound), consider them not simply as “medical images” but as streams of data generated by digital sensors requiring algorithmic interpretation.

The success of deep learning methods in interpreting complex medical signals and images in humans (e.g., automated ECG analysis [64] or high-precision diagnosis of diabetic retinopathy [65] and cataracts [66]) demonstrates the high maturity and competence of the algorithms. Signals obtained from diagnostic equipment are streams of data generated by digital sensors requiring algorithmic interpretation. This technological foundation serves as a benchmark for the adaptation of similar approaches to veterinary medicine. The transfer of these intelligent data analysis methods (in particular, convolutional neural networks, CNNs) enables the automation of diagnostic tasks such as contactless monitoring of physiological parameters or veterinary image analysis, which is crucial for precision animal husbandry and the timely detection of pathologies.

These technologies are also finding application in veterinary medicine: Park [67] describes the use of convolutional neural networks for automated screening of corneal opacities and cataracts in dogs using ultrasound images.

A similar high performance of deep neural networks (DNNs) is demonstrated in human oncology for classifying malignant skin lesions [68] and developing new biomarkers based on histological specimen analysis [69], where the algorithms reach the level of expert dermatologists. In the field of neurodegenerative research, convolutional (CNN) and recurrent (RNN) neural networks have been successfully applied for the early detection of Parkinson’s [70] and Alzheimer’s diseases [71] by analyzing gait parameters from wearable sensors or serial MRI brain scans. These successes in diagnosing complex pathologies in humans confirm the potential for transferring deep learning methods (DNNs, CNNs, RNNs) to veterinary science and animal husbandry to solve comparable problems: automatic recognition of hidden visual markers of diseases, analysis of histological data, and classification of behavioral anomalies based on data from wearable sensors (accelerometers), which is critical for proactive health management of farm animals.

**Figure 9:**
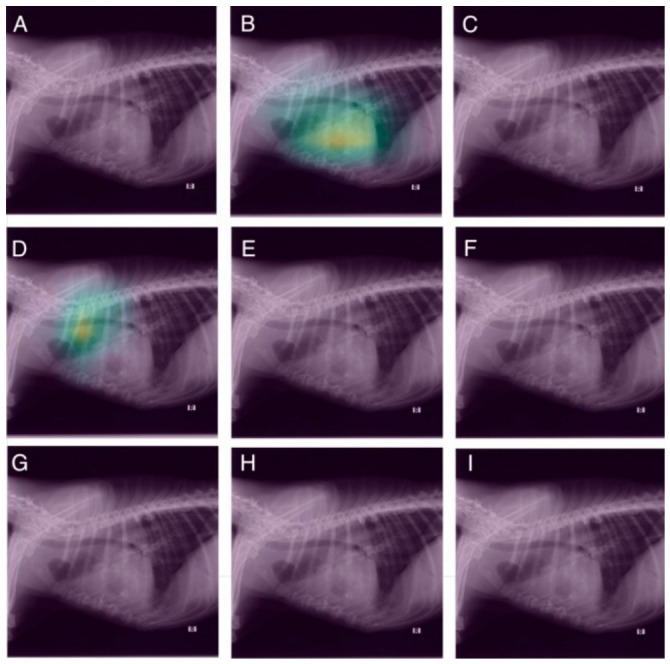
Visualization of the classification results performed by the ResNet-50 model using a canine chest radiograph with identified alveolar ridges in the cranial lobe [73]: (**A**)—Original image, (**B**)—alveolar pattern, (**C**)—bronchial pattern, (**D**)—cardiomegaly, (**E**)—mass, (**F**)—interstitial pattern, (**G**)—pleural effusion, (**H**)—pneumothorax, (**I**)—unremarkable.

In veterinary practice, deep learning (DL) addresses the challenges of improving animal productivity and monitoring animal welfare. Automated diagnostics are being actively implemented in radiology and ophthalmology. For example, Salvi et al. [72] developed a classification system for round cell tumors in dogs: a CNN analyzes tissue fragments selected by nuclear density and makes a final verdict using the “majority voting” method. Also, Banzato et al. [73] demonstrated the capabilities of automatic analysis of chest radiographs. The system calculates the vertebral heart score (VHS) for the diagnosis of cardiomyopathy. Despite the high accuracy in determining the alveolar pattern, the authors note the risk of false positives when searching for space-occupying lesions. Figure 9 shows a visualization of the classification results performed by the ResNet-50 model using the example of a dog’s chest radiograph with an identified alveolar pattern in the cranial lobe [73]. Colored highlights (heat maps) highlight specific anatomical zones: in image B, the heart region (cardiac silhouette) and adjacent central structures are highlighted in greenish-yellow; in image D, the heat map is shifted craniodorsally relative to the cardiac silhouette and highlights the cranial mediastinum, the base of the heart, and the projection of the major arteries in the tracheal bifurcation region.

Boufenar et al. [74] used deep learning to diagnose hip dysplasia in dogs, including German Shepherds. Their method allowed them to diagnose the condition with 98.32% accuracy. Spiteri et al. [75] used it to automatically detect syringomyelia (a severe neurological disorder) in Cavalier King Charles Spaniels using MRI images. Activations of the final layer are visualized by superimposing them on the radiographs.

Banzato et al. [76] presented a unified deep learning architecture capable of complex monitoring of canine and feline abdominal radiographs. The system simultaneously identifies various pathologies, including neoplasms, free fluid, and foreign objects. Deep learning technologies are also finding application in laboratory research automation: Morissette et al. [77] developed a specialized CNN-based platform for highly accurate evaluation of peripheral blood smears. The system automatically performs differential white blood cell counts, platelet analysis, and other cell populations. Concurrently, convolutional neural networks are being developed for rapid detection and species classification of parasites. The study of parasite detection (eggs and oocysts) in digital images of fecal samples from domestic animals (dogs, cats, and horses) is described in the article by Kumar et al. [78].

Lameness is a key indicator of welfare and losses in dairy farming. Sohan et al. [79] used video analysis with spatiotemporal deep learning to directly detect lameness in cattle. This paper describes the application of deep learning to the analysis of 3D cattle gait data obtained from cameras for the early prediction of lameness, a serious animal health and welfare issue.

Jermeikaite et al. [80] proposed a holistic model for detecting lameness in dairy cattle, integrating multimodal data, from physiological and behavioral patterns to the biochemical composition of blood and milk. To predict inflammatory and metabolic conditions, the authors successfully applied random forest and logistic regression algorithms. Automated diagnostics, implemented in a number of studies, are of strategic importance in poultry farming [81,82,83,84]. The main emphasis is on noninvasive methods: the use of convolutional neural networks (CNN) for analyzing feces images, as well as oral and cloacal swabs, which enables the detection of pathologies at early stages. Issues of rapid laboratory diagnostics are discussed in the works of Siddique [85], where AI (SVM, KNN, and Keras models) is used for rapid hematocrit analysis and the detection of anemia in small ruminants in the field. In addition, predictive algorithms such as IDEXX DecisionIQ [86] for assessing the risk of hyperthyroidism based on T4 and alkaline phosphatase levels, as well as decision tree-based models [87] for differentiating Addison’s disease based on standard blood test tabulations, are being introduced into clinical practice.

Proactive diagnosis of subclinical problems (metabolic stress, deficiency) by monitoring blood biochemistry using neural networks (e.g., Verax™) is described in [88]. Machine learning methods for animal disease classification are presented in Table 9.

Microwave technologies (radiometry, RF sensing) are a promising non-invasive diagnostic method in veterinary medicine; however, their widespread implementation requires solving technical problems borrowed from human medicine. In particular, the need to suppress powerful narrowband interference in highly sensitive biomedical receivers [89,90], which is critical for contactless monitoring of physiological processes, directly correlates with the task of isolating weak useful signals in the complex conditions of livestock complexes.

A direct logical connection with intelligent methods (ML, DL) is established at the data processing stage. Multimodal analysis of noisy signals received from microwave sensors is impossible without the use of algorithms such as random forest or neural networks [91,92]. These algorithms are used to: (1) filter external electromagnetic interference; (2) isolate diagnostic biomarkers (reflection, absorption, scattering); (3) classification of animal conditions, such as objective assessment of anemia in goats [91] or early detection of latent inflammatory processes by analyzing tissue thermal radiation [92].

Thus, microwave technologies integrated with machine learning models provide a proactive and objective approach to animal health management, allowing the detection of metabolic stress, nutrient deficiencies, or latent lameness at stages inaccessible to visual inspection [92].

This section of the review demonstrates that the sources [64,65,66,67,68,69,70,71] pertain to human medicine. The primary focus is on the automation of complex medical image analysis (ECG, fundus imaging, MRI), where AI approaches the level of experienced physicians (Table 10). Small animal veterinary medicine is described in [67,72,73,74,75,76,77], where AI successfully replicates medical approaches for diagnosing specific canine and feline ailments (orthopedics, endocrinology). Farm animals and their diagnostic methods are presented in [78,79,80,81,82,83,84,85,86,87,88,89,90,91,92]: Unlike human medicine, remote and physical methods (radiometry, lasers, Big Data sensors) predominate here, as it is important to monitor the health of large groups of animals without constant contact with each individual. Animal physiotherapy and [93] are represented by laser technologies, which stand out as a universal tool applicable to all animal species for the treatment of wounds, mastitis, and anemic surgeries.

A critical comparison of AI methods for disease diagnosis and animal health monitoring is provided in Table A3.

### 3.4. Analysis of Results

The analysis of heterogeneity among the evaluated studies revealed that algorithmic performance variations were primarily driven by the deployment environment and data scale. Models trained and tested solely on controlled, laboratory-derived datasets demonstrated inflated accuracy rates (often >95%), whereas systems deployed in real-world farm conditions or validated on independent external datasets exhibited a performance drop of 10–15% due to environmental noise, motion artifacts, and varying lighting conditions.

The sensitivity analysis confirmed the robustness of the review’s core qualitative conclusions. Excluding lower-quality studies—specifically those relying on simulated data, small non-representative datasets, or lacking any form of validation—did not alter the synthesized findings regarding the high maturity of computer vision for behavior tracking, nor did it shift the identified gaps regarding the need for multi-modal sensor fusion in industrial precision livestock farming.

The certainty of evidence varied across the evaluated technological outcomes. High certainty was established for behavioral tracking and microclimate monitoring due to extensive validation across diverse farm scales. Conversely, the evidence supporting early disease diagnosis and automated feed quality prediction was graded as low to moderate, downgraded primarily due to small, non-representative datasets, a lack of independent external validation, and low Technology Readiness Levels (TRLs).

The assessment of reporting bias across the thematic syntheses indicated a low risk of selective outcome reporting for established computer vision applications, as performance metrics were uniformly disclosed. However, a moderate risk of reporting bias was identified in emerging domains, such as real-time physiological profiling and multi-modal sensor fusion, where authors frequently omitted system failures, hardware connection losses, or poor algorithmic accuracy under edge-case conditions.

Several studies initially appeared to meet the inclusion criteria but were subsequently excluded upon full-text review. For instance, the study published in Sensors on autonomous vehicle optimization for cattle monitoring [94] initially appeared to meet the criteria due to its use of neural networks in a livestock setting. However, it was excluded upon full-text review because its primary focus was on vehicle routing logistics and robotic navigation paths rather than animal biomedical engineering, health diagnostics, or behavior profiling.

Additionally, the study exploring the applications of Radio-Frequency Non-Destructive Techniques (RF-NDT) in precision livestock farming [95] was excluded upon full-text evaluation. Although it fell within the domain of advanced technological monitoring for animals, it was excluded because it focused strictly on the radiophysical and dielectric properties of biological tissues rather than the deployment of automated artificial intelligence, computer vision, or machine learning frameworks for data synthesis.

This systematic review focuses on qualitative technological and engineering criteria, specifically the evaluation of artificial intelligence architectures and sensor configurations in precision livestock farming. Since the included studies did not involve randomized controlled clinical trials, traditional clinical risk of bias assessments are not applicable.

The synthesis conducted in this study is not statistical in nature. Primary studies were grouped based on their technological application, making a structured summary of risk of bias profiles for quantitative syntheses irrelevant within this framework.

Due to the high technical and methodological heterogeneity of the primary literature, which utilizes various neural network models, non-standardized evaluation frameworks, and different sensor types, we did not conduct a quantitative meta-analysis in this study. Consequently, the calculation of statistical summary estimates, confidence intervals, or mathematical measures of heterogeneity was not possible, and the results are presented solely through comprehensive thematic analysis and comparative matrices.

## 4. Discussion and Prospects

### 4.1. Discussion of Results

While human medicine [64,65,66,67,68,69,70,71] and small animal veterinary medicine [67,72,73,74,75,76,77] primarily focus on automating the analysis of complex medical images (ECG, MRI), industrial animal agriculture [78,79,80,81,82,83,84,85,86,87,88,89,90,91,92] is dominated by non-contact and physical methods for monitoring the health of large groups of animals. In this context, laser technologies [93] stand out as a universal tool for physiotherapy and surgery, applicable to all animal species for wound treatment, mastitis, and bloodless surgeries (including complex interventions such as neurosurgery or brachiocephalic syndrome correction [93]).

A comparison of intelligent methods by technology readiness level (TRL) and quality of evidence is presented in Table 11.

### 4.2. Prospects for Integrating Physical and Intelligent Methods

Laser therapy and surgery are also used in veterinary medicine. The author, Biletsky [93], a practicing veterinarian, demonstrated the potential of using the Lika-Surgeon+ laser device (manufactured by Photonika-Plus, Cherkassy, Ukraine) for treating animals, in particular: Skin incisions; Dental surgery; Laser surgery for brachiocephalic syndrome correction (rhinoplasty, soft tissue resection, laryngeal, choanal, and nasal surgery); New organ resection (endoscopic and open surgical methods); Endoscopic surgery for hard and soft tissue; Neurosurgery, etc.

We see the synergy of the physical capabilities of lasers [93] and intelligent control methods based on AI as a key area of promising research. This includes the development of computer vision-guided robotic laser systems for automatic tissue recognition and precision surgery, as well as the use of expert systems for automatic selection of laser therapy parameters based on multimodal data analysis (radiometry, sensors), which will enable the transition to fully autonomous and proactive animal health management.

Four areas can be identified here:

1 Computer vision-guided laser surgery (robotic surgery)
2 Smart laser therapy. Based on analysis of sensor data (e.g., tissue temperature, radiometry) and Big Data biomarkers, AI can adjust the laser dose in real time to achieve maximum therapeutic effect (e.g., in the treatment of mastitis or wounds [93]).
3 The creation of complex multimodal platforms combining AI-based intelligent diagnostics (e.g., veterinary image analysis [67,72,73,74,75,76,77]) and precision laser treatment [94]. AI can be used for automatic surgical planning, and lasers can be used as a high-precision tool for implementing this plan (e.g., in orthopedics or oncology).
4 Bioinformatics and laser photobiomodulation. The analysis of large volumes of metagenomic and transcriptomic data will allow AI to optimize laser therapy protocols at the molecular level.

Finally, this section is devoted to deep learning methods for interpreting medical data and proactive diagnostics. Medical image analysis includes the use of neural networks for analyzing X-rays, ultrasound, and MRI. This helps diagnose cardiovascular and oncological diseases. Multimodal analysis combines data from various sources (behavior, physiological indicators, video) to predict disease outbreaks before overt symptoms appear. Treatment technologies include laser systems. Lasers are used in surgery and therapy as a high-precision instrument.

## 5. Conclusions

The integration of intelligent technologies (ML, DL, Computer Vision, CNN) with advanced sensor systems (UWB, accelerometers, IoT) has found its way into PLF. Biomedical engineering and bioinformatics in veterinary medicine, combined with (PLF) and automated feed production, enable the creation of a comprehensive, data-driven ecosystem for managing animal health, nutrition, and behavior.

Improving feed quality and control: The use of ML and Computer Vision in non-destructive quality control of mineral and vitamin premixes eliminates the need for traditional laboratory methods. The use of hyperspectral imaging and ML models enables automated, rapid, and highly accurate assessment of mixing homogeneity, impurity detection, and stability prediction of key components (vitamins, carbonates, amino acids, etc.), which is crucial for maximizing feed biological efficiency and reducing environmental impact. Modern computer vision systems based on YOLO modifications demonstrate high efficiency in automatically tracking and classifying complex behavioral patterns (lying down, feeding, aggression, lameness) in cattle and pigs. When combined with ultra-wideband (UWB) positioning and accelerometers, this approach enables highly accurate positioning and early detection of behavioral anomalies that serve as indicators of subclinical diseases or stress.

Deep learning, particularly in the interpretation of medical images (radiographs, ultrasound) and the analysis of multimodal data (behavior, physiological parameters), is transforming veterinary medicine. This enables a shift from reactive treatment to proactive diagnosis of diseases, such as lameness in cows or orthopedic problems in small animals, at the earliest stages, significantly increasing the chances of successful recovery and reducing the use of antibiotics. Overall, intelligent systems not only automate existing processes but also provide farmers and veterinarians with new analytical tools for making informed decisions at the individual animal level. This leads to improved animal welfare, increased production efficiency, and the development of a more sustainable and environmentally responsible agricultural industry.

We see the following as key areas for further research: improving pattern recognition methods in animal husbandry and veterinary medicine through the implementation of new neural network models and their modifications; applying and testing new early diagnostic, therapeutic, and surgical devices on various animal species; creating a comprehensive information management system for real-world farming and agricultural holdings; standardizing data collection; and developing integrated platforms for seamlessly combining information from various sensor sources, cameras, and biomedical devices.

## Figures and Tables

**Table 1:** Distribution of articles by publisher and journal.

Publishing House/Platform	Number of Sources
MDPI (Animals, Applied Sciences, Foods, Agriculture, Sensors, Energies, AgriEngineering, Sustainability, Vet. Sci.)	33
Elsevier (Animal Feed Sci. & Tech., Food Control, J. Stored Products Research, Smart Ag. Tech., Research in Vet. Sci., Computers and Electronics in Ag., Computers in Bio. and Medicine, Data Brief)	11
Springer Nature (Scientific Reports, Nature, Nature Medicine, Multimedia Tools and Apps, Iran Journal of Computer Science, Arch. Comput. Methods Eng., Phys Eng Sci Med)	10
Frontiers Media (Frontiers in Veterinary Science, Frontiers in Nutrition, Frontiers in Artificial Intelligence, Frontiers in Robotics and AI)	6
American Dairy Science Association/Elsevier (Journal of Dairy Science)	4
IEEE (IEEE Access, IEEE Conference DESSERT)	2
PeerJ	2
Oxford University Press/ASAS (Journal of Animal Science and Technology)	1
Taylor & Francis (World’s Poultry Science Journal, Systems Science & Control Engineering, Computer Methods in Biomechanics)	3
Wiley (Journal of Veterinary Internal Medicine)	1
American Medical Association (JAMA)	1
American Veterinary Medical Association (AJVR)	1
Begell House (Telecomm. Radio Eng.)	1
IOS Press (Intelligent Systems and Computer Technology)	1
Science and Knowledge Research Society (IJACSA)	1
Siendo	1
Others/Web Resources/Preprints (Research Square, Quality Mag, Scorpion Vision, IDEXX, DSM-Firmenich, Fotonika Plus, Salud Ciencia y Tecnología, Animal Husbandry Products)	8

**Table 2:** Publications by Author Affiliation Country and Research Topics.

Country	Number of Articles	Main Topics/Examples
China	18	AI in animal husbandry, cow behavior monitoring, computer vision.
Italy	9	Deep learning in veterinary diagnostics, ultrasound analysis, sensors.
USA	8	Machine learning in medicine (Nature Medicine), cattle nutrition.
India	6	AI algorithms, milk quality, radiometric systems.
South Korea	5	Sensor technologies, sustainable development, data analysis.
Ukraine	4	Radio communication, cow productivity, electronics
Australia	3	Smart farms, wearable devices for animals
United Kingdom	3	Animal behavior analysis, computer vision.
Belgium	3	Milk data analysis, precision livestock farming
Canada	2	Grain storage technologies, disease diagnostics.
Germany	2	Sensors, oncology diagnostics.
Poland	2	Energy in the agricultural sector, data analysis.
Greece	2	Machine learning in the food industry.
Others (under 1 article)	8	Algeria, Lithuania, Nepal, the Netherlands, Slovakia, Tanzania, Thailand, Ireland.

**Table 3:** Publications by Research Center Country and Research Methodology.

Country	Number of Articles	Key Topics
China	22	Cow behavior (YOLO, CNN), pig disease detection, rice and honey quality.
USA	14	Deep learning in medicine (Nature Medicine, JAMA), canine and feline diagnostics (IDEXX, AJVR), animal nutrition.
Italy	10	Veterinary diagnostics (ultrasound, X-ray), buffalo mastitis, ultra-wide band monitoring systems.
South Korea	7	Machine vision for livestock monitoring, wearable sensors, pig health.
India	6	AI in agriculture, parasite classification, disease recognition.
Ukraine	4	Radiometric systems, electronics, cow productivity, laser surgery.
Australia	3	Behavior recognition using accelerometer data (CSIRO).
United Kingdom	3	Cancer biomarkers, calf behavior monitoring (UWB).
Canada	3	Grain storage technologies, poultry feed production.
Greece	3	Machine learning in the food industry, avian disease diagnostics.
Poland	2	Energy consumption in food systems (Visegrad countries).
Belgium	2	Cow behavior, farm automation.
Germany	2	Sensor systems, veterinary research.
Lithuania	1	Models for detecting lameness in cows.
Tanzania	1	Diagnosing poultry diseases using deep learning.
Colombia	1	Cost optimization in poultry farming (neural networks).
Others/Transnational	3	Research by DSM-Firmenich, Scorpion Vision, and others.

**Table 4:** Inclusion/exclusion criteria for literature in the review.

Criteria	Inclusion	Exclusion
Period	2015–2025	Before 2015
Publication Type	Journal articles, conference proceedings, books	Abstracts, preprints, patents, master’s theses (except when they contain unique technical data), web resources (except for scientific articles on the websites of specialized companies)
Language	English, Ukrainian, Russian	Chinese without an English abstract, all other languages
Subject	AI, neural networks, sensors in livestock farming, feed production, and veterinary medicine, sensor data analysis	Traditional veterinary medicine without automation, traditional animal husbandry, microclimate automation
Object	Farm animals, poultry, pets, feed and feed raw materials, veterinary medicine	Wild animals, rodents, fish, bees, laboratory animals, human medicine (except for Section 5, where they are used to compare the effectiveness of AI)
Methods and Technologies:	Use of AI methods (Machine Learning, Deep Learning, Computer Vision, CNN, YOLO, RNN, LSTM), modern sensor systems (UWB, accelerometers, gyroscopes, IoT), biomedical engineering (radiometry, laser therapy/surgery), and bioinformatics (metagenomic data analysis).	Human visual observation, manual data collection, traditional veterinary diagnostics, classical feeding rationing, automated systems without AI, telemetry and data recording without machine learning and classification algorithms, statistical methods without machine learning, irrelevant or outdated technical means, theoretical and conceptual models without validation
Databases	Scopus, WOS, Google Scholar	Articles without full access, duplicates

**Table 8:** Comparison of computer vision methods and UWB sensors for various criteria for animal monitoring.

Comparison Criteria	Computer Vision	Ultra-Wideband Sensors (UWB/RTLS)
Technical Advantages	Contactless—no need to attach sensors to the animal, eliminating the need for maintenance (battery replacement) [63] (RFR-YOLO). High information content—allows for detailed classification of complex behavioral patterns (feeding, chewing, aggression, breathing, body position) [38,41,42,54].	High positioning accuracy: provides precise animal location coordinates within the barn [44,45]. Lighting independence: stable operation in all lighting conditions [48].
Technical Disadvantages and Limitations	Sensitivity to the environment—accuracy decreases due to changes in lighting [33], dust, or occlusion of animals in dense groups [39,40,61].	Structural influence: metal barn elements cause signal reflection and create “blind spots” [44,62]. Data noise: raw data requires complex software filtering and smoothing [46].
Complexity of Deployment on a Farm	Medium—requires installing cameras with overlapping fields of view [61] and ensuring high-quality lighting [38].	High—requires complex installation of stationary antennas (anchors) around the perimeter [45,62]. Requires individual tagging of each animal.
Operating Costs (Maintenance)	Low—cameras operate from a fixed network; no animal maintenance is required [42].	High—requires regular battery replacement in animal tags [42].
Computing Resources	High—real-time video stream processing requires powerful graphics processing units (GPUs) [34] or optimization of models for Edge-AI [18]. Video stream, semantic segmentation, action classification (lying, standing, feeding, aggression, respiration) [38,41,42,55].	Low/Medium—processing numerical coordinate data is less resource-intensive, focusing primarily on noise reduction algorithms [46]. Time series of 2D/3D coordinates (X, Y, Z), time spent in the zone (feeding/lying zone) [44,62].
Type of Data Obtained	Computer Vision	Ultra-wideband sensors (UWB/RTLS)

**Table 9:** Machine learning methods for animal disease classification.

Biological Object	Methods	Examination Results	Source
**Pets**			
Dogs	DeepLensNet, DenseNet-161	Cataracts Based on Ultrasound Images of the Eyeball	[67]
Dogs	CNN	Histopathological classification of tumors in dogs (round cell)	[72]
Dogs	CNN	Estimation of canine heart size from chest radiographs	[73]
Dogs	CNN	Detection and assessment of elbow dysplasia on standard radiographs	[74]
Dogs	CNN	Syringomyelia (a neurological disorder) using MRI images	[75]
Cats, dogs	DL	Simultaneous detection of multiple pathological changes (foreign bodies, tumors, fluid collections) on abdominal radiographs	[76]
Cats, dogs	CNN	Evaluation of peripheral blood smears, including automated differential counts of leukocytes, platelets, and other cells	[77]
Cats, dogs, horses	CNN	Detection and classification of parasites (eggs and oocysts) on digital images of fecal samples	[78]
Cats	IDEXX DecisionIQ	Classification of Addison’s disease based on complete blood count and serum biochemistry	[86]
Dogs	Decision trees and other ML models	Proactive diagnosis of subclinical problems (metabolic stress, deficiency) by monitoring blood biochemistry	[87]
**Farm animals and poultry**			
Cattle	DL, 3D data analysis	Gait assessment for early prediction of lameness, a serious health and welfare issue for animals. Prediction of lameness based on blood biochemistry parameters	[79]
Cattle	Random forest, logistic regression	Analysis of cloacal, oral, and fecal images	[80]
Birds	CNN	Detection of anemia by analyzing blood sample images (PCV value) using models	[81,82,83,84]
Ruminants	SVM, KNN, and Keras	Prediction of hyperthyroidism risk based on biochemistry parameters (T4, ALP) and other clinical data	[85]
Birds	CNN	Examination Results	[88].
**Samples for comparison with humans**			
	CNN	Types of Cardiac Arrhythmias Based on Ambulatory Electrocardiograms (ECGs)	[64]
	CNN	Diabetic Retinopathy in Retinal Photographs	[65]
	CNN	Diagnosis and Quantitative Classification of Cataract Type and Severity	[66]
	DNN, CNN	Skin Cancer Classifications (Melanoma and Squamous Cell Carcinoma) Based on Images	[68]
	DNN	Integration of Pathological Images with Cancer Genomic Data	[69]
	CNN, wearable sensors	Detection of Early Stage Parkinson’s Disease Based on Walking Test Data Using Gait Analysis (Gait)	[70]
	RNN	Classification of Patients with Alzheimer’s Disease (AD) and Mild Cognitive Impairment Based on Brain MRI Sequences	[71]

**Table 10:** Biomedical engineering methods related to specific animals or humans.

Research Object	Source	Technologies Used	Key Result
Dogs	[67,72,73,74,75,77,92]	CNN (DeepLensNet), ML algorithms, radiography, laser surgery.	Diagnosis of cataracts, tumors, liver diseases, joint dysplasia, and Addison’s disease. Laser removal of tumors.
Cats	[76,92]	CNN, laser photobiomodulation.	Automated diagnostics of hyperthyroidism. Use of lasers for pain relief and inflammation treatment.
Cattle	[83,84,92]	Physiological parameter sensors, laser therapy.	Herd health monitoring. Effective treatment of mastitis and stimulation of reproductive function with lasers.
Horses	[86,93]	Laser therapy.	Treatment of musculoskeletal pathologies and accelerated wound healing.
Farm Animals (General Systems)	[78,89,91]	Big Data, biomarkers, microwave radiometry.	Early warning systems for gastrointestinal diseases. Remote detection of internal inflammation using thermal radiation.
General Methods (Animals and Medicine)	[79,80,81,82,85,87,88]	IoT, RF sensing, telemedicine, mobile apps.	Development of non-invasive health monitoring methods (pulse, temperature), infection outbreak prediction, laser ablation.
Samples for comparison with humans	[64,65,66,67,69,70,71]	Deep learning (CNN, DNN, RNN), ECG, MRI, and CT analysis.	Highly accurate diagnostics of arrhythmias, retinal pathologies, skin cancer, and neurodegenerative diseases (Parkinson’s, Alzheimer’s).

**Table 11:** Comparison of intelligent methods by technology readiness level (TRL) and quality of evidence.

Intelligent Method or Technology	Applications in Livestock	Technology Readiness Level (TRL)	Quality of Evidence
Computer Vision (models such as YOLO, ResNet)	Monitoring of activity, feeding, and chewing [38,41], behavior classification (lying, standing) [38,42], and welfare assessment [54,55].	TRL 7-9 Production Implementation, Commercial Systems	High: Numerous long-term field trials on real commercial farms [38,42,61]. Use of large, representative datasets (>10,000 images or videos) [38,61]. External validation on independent herds.
Wearable Sensors (accelerometers, UWB)	Location tracking (RTLS), activity monitoring (lying time, steps) [44,45], and early lameness diagnosis [46].	TRL 7-9 Commercial Systems, Widespread Use	High: Long-term field trials on real commercial farms [44,45,62]. Use of large time series datasets from hundreds of animals [44]. External validation of noise reduction and classification algorithms [46].
NIR Spectroscopy + AI (ML/DL)	Non-destructive quality control of feed (amino acids, vitamins) [33,34].	TRL 6-8 Prototypes, Pilot Operation	Medium/High: Field validations in feed mills and farms [33]. Use of large spectral datasets [34]. However, the quality of evidence is limited by the high complexity of model calibration and sensitivity to environmental conditions [30].
Raman Spectroscopy + AI (ML/DL)	Rapid detection of adulteration and confirmation of the authenticity of feed additives (amino acids) [24,29].	TRL 4-6 Laboratory Research, Prototypes	Medium: Predominantly laboratory studies under controlled conditions [5,34,78]. Use of relatively small datasets. Lack of external field validation in real production [19,24].
Microwave Technologies (RF Sensing, Radiometry)	Early diagnosis of anemia (goats) [8] and latent inflammation (mastitis, lameness) through thermal radiation analysis [92].	TRL 4-6 Laboratory Research, Prototypes	Moderate: Predominantly laboratory studies (vivarium, experimental farm) under controlled conditions [90]. Use of small animal samples [91]. The quality of evidence is limited by the high sensitivity of real farms to electromagnetic interference [89] and the lack of external field validation.
Bioinformatics + Big Data Analytics	Metagenomic data analysis for feed optimization and productivity prediction [22,74].	TRL 3-5 Conceptual Research, Laboratory Prototypes	Low or Moderate: Predominantly conceptual and laboratory studies [17,69]. Use of large datasets, but often without linkage to phenotypic data of real animals. Low level of external validation of models in real production.

## Data Availability

The data presented in this study are available on request from the corresponding author.

## Supplementary Materials

The following supporting information can be downloaded at https://www.mdpi.com/article/10.3390/s26134015/s1, Table S1: PRISMA 2020 Main Checklist.

## Appendix A

**Table A1:** Critical comparison of intelligent methods for feed and premix quality control.

Task—Feed Quality Control	Sensor/Method (Technology)	AI Model	Ground Truth	Metrics	Deployment: Lab/Plant	Failure Modes & Limitations	References
Rapid analysis of amino acids in raw materials	NIR spectroscopy (Hyperspectral)	Partial Least Squares (PLS)/ML	Chemical Analysis (HPLC)	R-squared, RMSEP (Root Mean Square Error of Prediction)	Field (Plant): Feed mill, actual batches	High complexity of model calibration, requiring extensive heterogeneous datasets. Sensitivity to humidity and sample temperature.	[33]
Vitamin concentration in premixes	NIR spectroscopy	Deep Learning	Chemical Analysis (HPLC)	Accuracy, RMSE	Laboratory (Lab): Pilot production	Need for data augmentation to prevent overfitting of deep learning models.	[8]
Detection of adulteration	Raman spectroscopy	SVM + Random Forest (RF)	Chemical Analysis/Gas Chromatography	Accuracy, F1-score	Laboratory (Lab): Controlled conditions (honey example)	High equipment cost compared to NIR. Sensitivity to fluorescence of complex samples. Lack of external field validation	[21,26,34]
Vitamin A stability	Chemical analysis (non-AI, basic research)	N/A (Kinetic Modeling)	Chemical Analysis (HPLC)	% Retention	Laboratory (Lab): Controlled storage conditions	Basic stability study that does not use AI methods for prediction.	[15]

**Table A2:** Critical comparison of AI methods for animal behavior recognition.

Task—(Behavior)	Sensor	AI Model	Ground Truth	Metrics	Deployment: Lab/Farm	Failure Modes & Limitations	Ref.
Cattle behavior classification (lying, standing, feeding)	Video camera (RGB)	YOLOv5 + Res-Dense	Manual Video Annotation	Accuracy, Precision, Recall	Field (Farm): Real barn, 10 cows	Sensitivity to light, animal overlap in dense groups.	[38]
Respiration rate	Thermal camera (Thermal)	CNN (G-CNN-LSTM)	Spirometry/Manual Scoring	MAPE (Mean Absolute Percentage Error)	Laboratory (Lab): Vivarium, 5 cows	High sensor cost, impact of ambient temperature.	[41]
Aggression detection	Video camera (RGB)	Improved YOLO.X	Manual Video Annotation	mAP (mean Average Precision)	Laboratory (Lab): Experimental pen	Difficulty detecting fast movements, pig overlap at feeders.	[55]
Activity and location tracking (RTLS)	UWB sensor (wearable tag)	SVM + Random Forest	Visual Observation/GPS	Position Error, Accuracy	Field (Farm): Commercial farm, 100 cows	Influence of metal farm structures on the UWB signal, noise in the data (requires smoothing).	[45]
Lameness detection	Accelerometer + Gyroscope	CNN-LSTM	Visual Scoring	Sensitivity, Specificity, F1-score	Field (Farm): Real farm, 30 cows	Difficulty detecting early lameness, gait variability in animals.	[41]

**Table A3:** A critical comparison of AI methods for disease diagnosis and animal health monitoring.

Name of Method II/Model	Research Object	Results	Source Numbers
CNN (DeepLensNet, DenseNet-161)	Dogs	Cardiac arrhythmia types based on ambulatory ECGs; diabetic retinopathy; diagnosis and classification of cataracts in humans and dogs using ultrasound images	[64,65,66,67]
CNN, Deep Learning (DL)	Dogs, Cats	Histopathological classification of tumors; cardiac size assessment using radiographs; detection of elbow dysplasia and syringomyelia; multipathological analysis of abdominal images	[72,73,74,75,76]
CNN, Image analysis, Expert systems	Dogs, Cats, Horses	Automated differential blood cell count; parasite detection in fecal samples; classification of feline Addison’s disease (IDEXX DecisionIQ)	[77,78,86]
DL, 3D data analysis, ML algorithms (Random Forest, LogReg)	Cattle	Gait assessment of cattle for early prediction of lameness; analysis of mucous membrane and fecal images to assess inflammatory and metabolic conditions	[79,80]
CNN	Poultry	Anemia detection using digital blood fluoroscopy (PCV); non-invasive rapid diagnostics of diseases using smear and stool analysis in poultry farms	[81,82,83,84]
SVM, KNN, Keras, technological solutions	Ruminants, Dogs	Prediction of hyperthyroidism risk in ruminants; Proactive diagnostics of subclinical metabolic stress and deficiency states using blood biochemistry	[85,87]
CNN, Big Data, microwave radiometry, IoT, Thermal radiation	Poultry, Farm Animals	Diagnostics of poultry diseases; remote detection of internal inflammation in livestock using microwave radiometry; non-invasive monitoring of health parameters (pulse, temperature)	[88,89,90]
Telemedicine, Mobile Teleapplications, ML-algorithms	Farm Animals	Early warning systems for gastrointestinal diseases in livestock groups on farms; herd health monitoring	[91]
